# The Relationship Between Referral of Touch and the Feeling of Ownership in the Rubber Hand Illusion

**DOI:** 10.3389/fpsyg.2021.629590

**Published:** 2021-02-11

**Authors:** Arran T. Reader, Victoria S. Trifonova, H. Henrik Ehrsson

**Affiliations:** ^1^Department of Psychology, Faculty of Natural Sciences, University of Stirling, Stirling, United Kingdom; ^2^Department of Neuroscience, Karolinska Institutet, Stockholm, Sweden

**Keywords:** body perception, limb ownership, multisensory integration, referral of touch, rubber hand illusion

## Abstract

The rubber hand illusion (RHI) is one of the most commonly used paradigms to examine the sense of body ownership. Touches are synchronously applied to the real hand, hidden from view, and a false hand in an anatomically congruent position. During the illusion one may perceive that the feeling of touch arises from the false hand (referral of touch), and that the false hand is one's own. The relationship between referral of touch and body ownership in the illusion is unclear, and some articles average responses to statements addressing these experiences, which may be inappropriate depending on the research question of interest. To address these concerns, we re-analyzed three freely available datasets to better understand the relationship between referral of touch and feeling of ownership in the RHI. We found that most participants who report a feeling of ownership also report referral of touch, and that referral of touch and ownership show a moderately strong positive relationship that was highly replicable. In addition, referral of touch tends to be reported more strongly and more frequently than the feeling of ownership over the hand. The former observations confirm that referral of touch and ownership are related experiences in the RHI. The latter, however, indicate that when pooling the statements one may obtain a higher number of illusion ‘responders’ compared to considering the ownership statements in isolation. These results have implications for the RHI as an experimental paradigm.

## Introduction

Body ownership refers to the feeling that the observed body belongs to the self. This sensation is believed to arise through multisensory integration, whereby a combination of different sources of sensory information (vision, touch, proprioception, etc.) can give rise to a coherent percept of the body as one's own (Kilteni et al., 2015; Samad et al., 2015; Ehrsson, 2020). Manipulating multisensory information can therefore alter the feeling of body ownership, most notably during the rubber hand illusion (RHI) (Botvinick and Cohen, 1998), possibly the most frequently used multisensory body ownership illusion (Kilteni et al., 2015; Riemer et al., 2019). During the classic version of the RHI, touches applied to a false hand in spatial and temporal synchrony with the participant's real (hidden) hand can induce a feeling of ownership over the false hand. Asynchronous visuotactile stimulation of the two hands with a large temporal discrepancy does not induce a feeling of ownership over the false hand (Shimada et al., 2009; Costantini et al., 2016; Ismail and Shimada, 2016; Chancel and Ehrsson, 2020), and so this procedure is often used as a control condition.

The RHI is striking not just because of the feeling of ownership over a false limb, but also because of the referral of touch that can occur during illusion induction, where it seems like the felt touch is caused by the stimulus applied to the false hand. Some theoretical models of the RHI posit that referral of touch occurs prior to the feeling of ownership over the false hand (Makin et al., 2008; Valenzuela Moguillansky et al., 2013), potentially reflecting a causal relationship whereby referral of touch is necessary, but not sufficient, to induce the sense of ownership. Other neurocognitive models have suggested that referral of tactile sensation to the false hand gives rise to the subjective experience of body ownership (Tsakiris, 2010), which implies a very intimate connection between the two. Finally, alternative models conceptualize ownership and referral of touch as different components in a single multisensory integration process (Ehrsson, 2020) where referral of touch reflects visuotactile combination and ownership reflects multisensory causal inference (Samad et al., 2015; Ehrsson and Chancel, 2019; Fang et al., 2019; Litwin, 2020).

The degree to which participants report referral of touch, as well as the feeling of ownership over the false hand, are commonly used as subjective assessments of the strength of the illusion. In fact, some articles average (usually mean) these statements, to provide a single value representing the overall strength of the illusion (sometimes referred to as an “RHI index”). This RHI index is occasionally used to draw inferences about the influence of experimental manipulations on body ownership (e.g., Smit et al., 2017; Shibuya et al., 2018). However, referral of touch tends to be experienced more strongly and more frequently than ownership during synchronous tactile stimulation (Holmes and Spence, 2007; Kalckert et al., 2019) and participants may report referral of touch without expressing a feeling of ownership.

Ultimately, the fundamental relationship between referral of touch and body ownership remains unclear. Importantly, referral of touch seems to be more flexible than ownership, given possible occurrence in the absence of changes in body ownership perception. Most significant is perhaps the referral of touch to the tips of hand-held tools (e.g., Miller et al., 2018), and in the context of the RHI, referral of touch has sometimes been reported to non-corporeal objects in the absence of ownership. Notably, Kalckert et al. (2019) recently examined participant experiences of referral of touch and feeling of ownership when visuotactile stimuli were applied synchronously to the real hand and to either a false hand (RHI) or to a balloon. They did this to assess the implications of comparing responses to synchronous and asynchronous visuotactile stimulation as a measure of successful illusion induction. Importantly, they found that it was possible to induce a referral of touch to non-bodily objects in some individuals, even when ownership was not induced. Observations like these pose a challenge for models of body ownership that emphasize the importance of referral of touch. A related methodological point is that Kalckert et al. (2019) also showed that averaging referral of touch and ownership responses can increase the magnitude of ratings, even in scenarios not inducing a feeling of ownership. As noted by the authors, this means that different approaches to analyzing questionnaire data during the RHI might lead to different conclusions.

This latter point has implications for how questionnaire data in RHI studies are analyzed and interpreted. In particular, is possible that averaging referral of touch and ownership statements may only be appropriate if one is interested in testing the effects of experimental manipulations on the RHI itself (e.g., Abdulkarim and Ehrsson, 2016), rather than making any statement specifically about the subjective body ownership experience. If an experimenter is explicitly interested in using the RHI to test the effect of an experimental manipulation on body ownership specifically, or the effects of manipulating body ownership on a secondary variable, then averaging ownership and referral of touch statements may result in an overestimation in the strength of ownership experience. This may reduce the accuracy of the results, since ownership ‘non-responders’ (individuals that were in fact not affirming illusory ownership) would be used to draw inferences about body ownership.

We decided to perform a more detailed assessment of referral of touch and feeling of ownership in the RHI, assessing their relationship and relative response rate in order to better understand these phenomena and examine the methodological implications of combining the two statements. We re-analyzed data from a previous experiment (Reader et al., 2020) to examine the strength of subjective experience for referral of touch and ownership, characterize the degree of correlation between these experiences, and to see whether using an RHI index results in different reporting estimates. In addition, we re-analyse freely available datasets from two other articles to validate our findings, and perform a descriptive assessment of the relative experience of referral of touch and ownership and how replicable their relationship is.

## Method

We re-analyzed data from a previous experiment (Reader et al., 2020, experiment 2), which are freely available (https://doi.org/10.17605/OSF.IO/NYHZQ). The full experimental protocol is described in the previous article, but we provide a brief overview herein.

Our sample included 59 participants (6 left-handed, 30 female, mean ± SD age = 26.4 ± 5.63). Questionnaire responses to the illusion were recorded following the main experimental paradigm. Participants sat opposite the experimenter and placed their right hand behind a screen. A prosthetic right hand was placed to the left of the screen, aligned as closely as possible to the participant's right shoulder. The middle finger of the false hand and the middle finger of the real right hand were placed 20 cm apart, both 10 cm away from the screen. The participant's upper body and arms were covered with a black cloth as to occlude the gap between the false hand and the participants body. The false arm then appeared beneath the cloth visible to the participant in a forward pointing orientation so that it looked like it could be the participant's own limb. Tactile stimulation was applied to the real and false hands for 30 s using a brush, in counterbalanced synchronous and asynchronous conditions. After tactile stimulation, subjective experience during the RHI was assessed using questionnaire items that participants were requested to respond to on a 7-point Likert scale (−3: strongly disagree, +3: strongly agree). This included a single statement addressing the feeling of ownership and a single statement addressing referral of touch (Table 1).

**Table 1:** Questionnaire information and response rates in the synchronous condition.

**Article**	**Ownership statement**	**Referral of touch statement**	**Rating system**	**Positive response to referral of touch statement (%)**	**Positive response to ownership statement (%)**	**Positive response for RHI index (%)**	**Participants with RHI index value greater than ownership value (%)**
Reader et al. (2020)	“It seemed like the rubber hand was my hand”	“It seemed like the touch I felt was caused by the brush touching the rubber hand”	+3 (“strongly agree”) to −3 (“strongly disagree”)	73	58	64	58
Engelen et al. (2017)	“I felt as if the rubber hand were my hand”	“It seemed as though the touch I felt was caused by the finger touching the rubber hand”	1 to 7	85	76	83	64
Motyka and Litwin (2019)	“I felt as if the rubber hand were my hand”	“It seemed as though the touch I felt was caused by the paintbrush touching the rubber hand”	+3 (“fully agree”) to −3 (“fully disagree”)	66	66	68	36

In this re-analysis we compared responses to referral of touch and ownership statements within participants using a two-tailed Wilcoxon signed-rank test, with the effect size r given as the rank-biserial correlation. We also assessed the correlation between responses to the two statements using Kendall rank correlation (tau-b) in the synchronous condition, as well as correlating the difference between synchronous and asynchronous responses for the two statements (to account for individual response criteria and general cognitive bias). We then examined positive responses to referral of touch and ownership in the synchronous condition (i.e., the condition that elicits the RHI). Positive responses were defined as affirming the subjective experience, reflected in a response >0. To create a combined measure of referral of touch and ownership (RHI index) we took the mean value of the two statements. We then calculated the percentage of participants with a positive response for the RHI index, as well as the percentage of participants who had a greater value for the RHI index than the ownership statement alone.

To validate results observed in our own data, we sought openly available data from other researchers which we analyzed in the same fashion. We performed a search in Web of Science (https://www.webofknowledge.com) for items containing the term “rubber hand illusion” (all databases, search term TS = “rubber hand illusion”), published between 2017 and 2019 (search date 2nd September 2019). This returned 238 results of which 219 were classified as articles. We screened the abstracts, first excluding those which were reviews, meta-analyses, commentaries, conference proceedings, corrections, or experimental articles that did not use a visuotactile version of the RHI (*n* = 149). Articles were further excluded if they were testing a clinical population or children (*n* = 17). Next, articles were examined in detail and excluded if they did not provide freely available open data (*n* = 42). Remaining articles were further assessed, and any that did not report both referral of touch and ownership statements were also excluded (*n* = 4). Seven articles remained, of which two provided responses to individual questionnaire items (Engelen et al., 2017; Motyka and Litwin, 2019).

Engelen et al. (2017) employed a between-subject design to examine the influence of affective vocalizations on the RHI in 208 right-handed participants (*n* = 114 in experiment 1, 90 female, mean ± SD age = 22.5 ± 3 years; *n* = 94 in experiment 2, 69 female, mean ± SD age = 23.4 ± 5 years). Participants were asked to place their right hand 10 cm to the right side of a barrier on the table. A plastic false hand was placed 10 cm to the left of this barrier, aligned with the participants' shoulder, and a cloth covered both the false hand in front of them and their real hand up to the wrist. The first experiment had four conditions with different sounds delivered through the headphones during visuotactile stimulation (happy vocalization, angry vocalization, non-vocal, no sound). The second experiment had three conditions (angry vocalization, neutral vocalization, and no sound). Participants took part in three trials each of synchronous and asynchronous tactile stimulation, with each trial lasting 1.5 min. Subjective experience during each trial was assessed using a questionnaire that contained statements about false hand ownership and referral of touch (Table 1), with responses given on a scale from 1 to 7. For re-analysis, we combined the data from experiments 1 and 2, but only took data from those in the “no sound” conditions (*n* = 59). We calculated the mean questionnaire response to each statement for all trials. To create an RHI index we took the mean value of the complementary referral of touch and ownership statements. Positive responses were considered those >4, since this would be akin to the central 0 value in our data.

Motyka and Litwin (2019) used a between-subjects design with a sample of 50 right-handed participants split into two groups. They analyzed only 49 participants following an exclusion (33 female, mean ± SD age = 23.8 ± 3.7 years), though we made use of their entire dataset (i.e., *n* = 50) since the exclusion was based on reproduction errors in a proprioceptive accuracy task rather than subjective questionnaire responses. During the RHI component of their experiment, a model hand was placed on a platform in front of the participants, as if it were aligned with their shoulder, while their real right hand was hidden underneath the platform−12.5 cm (vertical) and 16 cm (lateral) away from the false hand. Then, the experimenter displaced the horizontal location of the right hand of the participants to one of two locations—either close (8 cm) or far (24 cm) from the false hand. Subsequently, the experimenter applied synchronous or asynchronous tactile stimulation to the real and the false hand for 2 min. Finally, the participants were presented with a 9-item questionnaire to measure the subjective strength of the illusion, which included items for referral of touch and body ownership (Table 1). For re-analysis, we combined the data from both groups of participants. To create an RHI index we took the mean value of the complementary referral of touch and ownership statements.

Note that since our dataset used only a single statement to address referral of touch, we used only the single complementary statement in the reanalysis of Engelen et al. (2017) and Motyka and Litwin (2019). However, another statement was used by these authors, as is common in RHI experiments. This statement typically reads “It seemed as if I were feeling the touch of the brush in the location where I saw the rubber hand touched.” To address this, we also performed an analysis of response rates using an RHI index made up of both referral of touch statements and the ownership statement (Supplementary Table 1), as is reported in some previous experiments (e.g., Abdulkarim and Ehrsson, 2016). Results showed a similar pattern as that described below. We also provide some comparisons between the two referral of touch statements for interested readers in Supplementary Material.

Finally, we assessed the proportion of people who affirm referral of touch and ownership to provide a description of individual subjective experience during the illusion. To make the most of the different datasets available, we combined our data with that of Motyka and Litwin (2019), who employed the same rating system. We also assessed responses in the asynchronous condition as a descriptive comparison, and provide the overlapping rates of affirmation to all conditions in Supplementary Material. With this larger dataset we performed two-tailed Wilcoxon signed-rank tests to examine effect sizes when comparing synchronous and asynchronous conditions with solely the ownership statement, solely the referral of touch statement, or with the RHI index.

## Results

**Figure 1:**
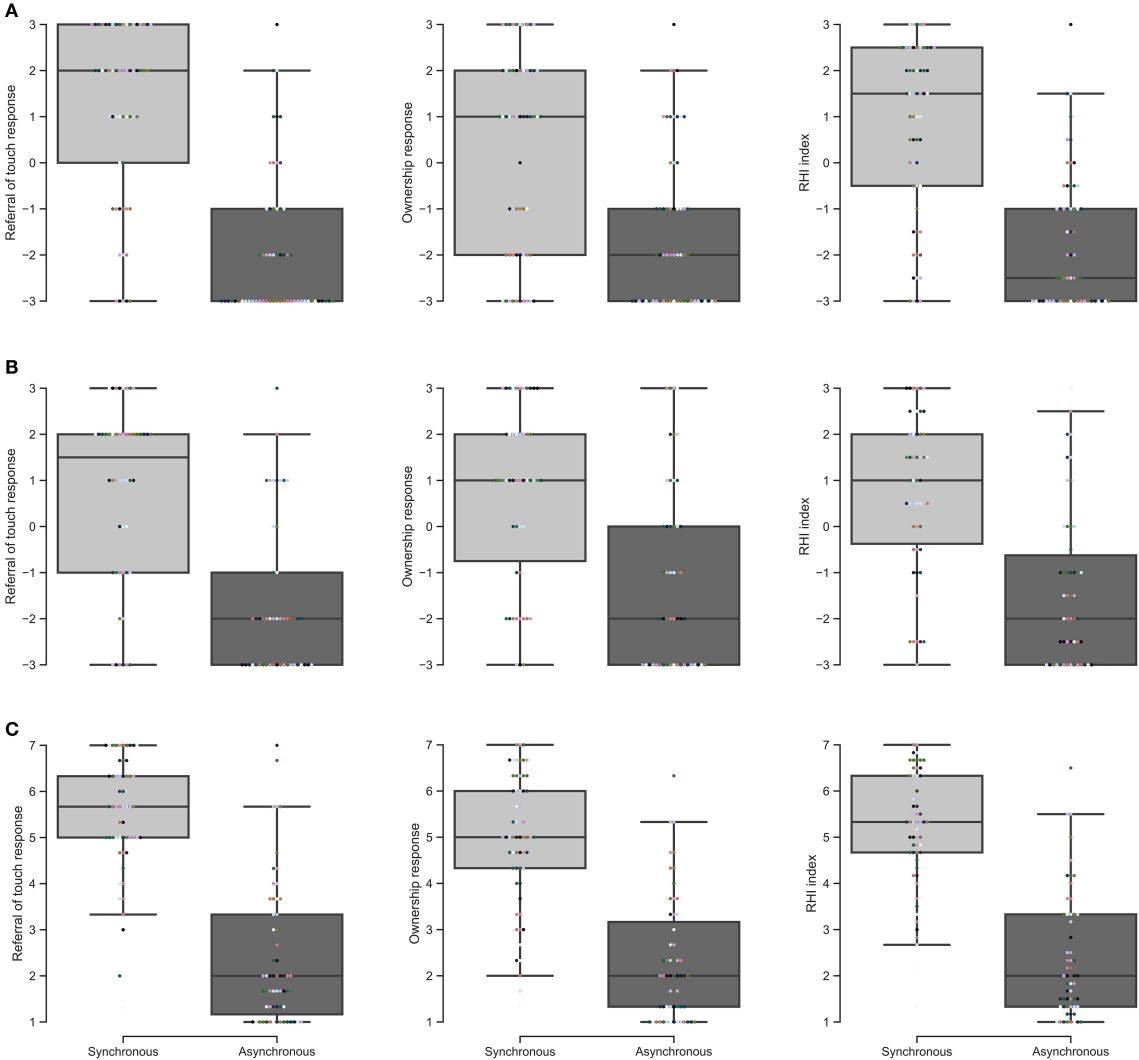
Questionnaire responses. **(A)** Reader et al. (2020), **(B)** Motyka and Litwin (2019), and **(C)** Engelen et al. (2017).

In our own data, we observed that participant responses to referral of touch were greater than those for ownership in the synchronous condition (medians = 2 vs. 1, *W* = 774, *p* < 0.001, *r* = −0.125, 95% CI = [−0.397, 0.166]), an effect observed in 58% of participants (Figure 1). The inverse effect was observed in the asynchronous condition (medians = −3 vs. −2, *W* = 105, *p* = 0.0395, *r* = −0.881, 95% CI = [−0.932, −0.796]), though this was only evident in 29% of participants, and 54% of participants provided an equal response for both statements. The difference between the synchronous and asynchronous conditions was greater for referral of touch than ownership (medians = 3 vs. 2, *W* = 969.5, *p* < 0.001, *r* = 0.0955, 95% CI = [−0.195, 0.371]), an effect observed in 64% of participants.

**Figure 2:**
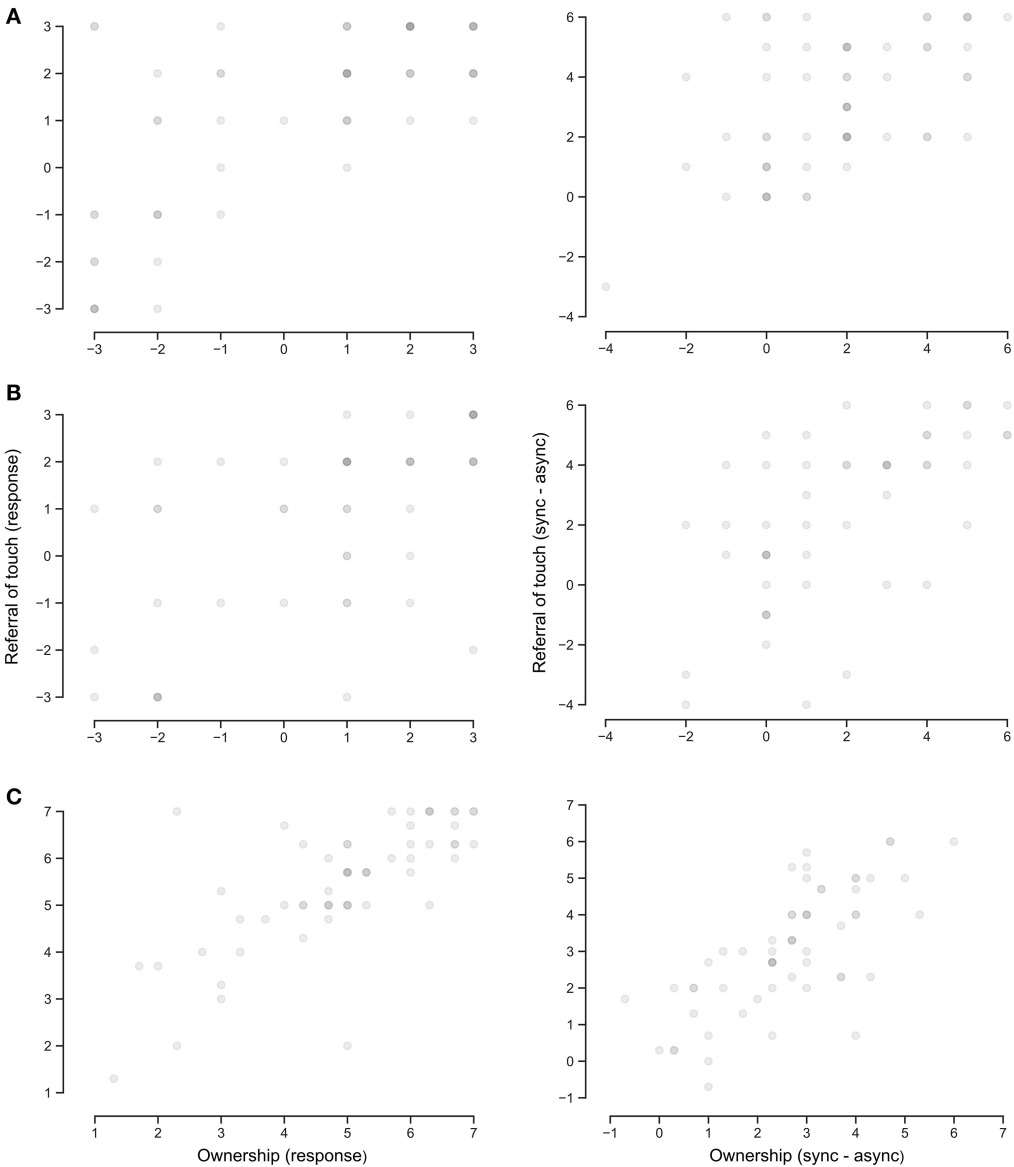
Scatterplots comparing responses to referral of touch and ownership in the synchronous condition (left) and with the difference between the synchronous and asynchronous conditions (right). Data are displayed for **(A)** Reader et al. (2020), **(B)** Motyka and Litwin (2019), and **(C)** Engelen et al. (2017).

Responses to the referral of touch and ownership statements in the synchronous condition were positively correlated (*r*_τ_ = 0.554, 95% CI = [0.401, 0.706], *p* < 0.001, Figure 2), as was the difference between synchronous and asynchronous statements (*r*_τ_ = 0.381, 95% CI = [0.207, 0.555], *p* < 0.001). The percentage of participants with a positive response to referral of touch was greater than that for ownership in the synchronous condition (73 vs. 58%) (Table 1). In addition, the percentage of participants with a positive response for the RHI index (64%) was greater than that for the ownership statement, and 58% of participants had an RHI index value greater than their response to the ownership statement. In comparison, only 17% of participants had an RHI index value greater than their response to the ownership statement in the asynchronous condition.

In the data presented by Engelen et al. (2017), responses to referral of touch were greater than those for ownership (medians = 5.67 vs. 5.00, *W* = 948.5, *p* < 0.001, *r* = 0.0718, 95% CI = [−0.218, 0.349]), an effect observed in 64% of participants (Figure 1). Similar results were not observed for the asynchronous condition (both medians = 2.00, *W* = 483, *p* = 0.909, *r* = −0.454, 95% CI = [−0.655, −0.194]). However, the difference between the synchronous and asynchronous conditions was greater for referral of touch than ownership (medians = 3 vs. 2.67, *W* = 967.5, *p* = 0.00435, *r* = 0.0932, 95% CI = [−0.197, 0.369]), an effect observed in 61% of participants.

Responses to the referral of touch and ownership statements were positively correlated in the synchronous condition (*r*_τ_ = 0.607, 95% CI = [0.459, 0.756], *p* < 0.001, Figure 2), and when examining the difference between synchronous and asynchronous conditions (*r*_τ_ = 0.580, 95% CI = [0.448, 0.711], *p* < 0.001). The percentage of participants with a positive response to referral of touch was greater than that for ownership (85 vs. 76%; Table 1). In addition, the percentage of participants with a positive response for the RHI index (83%) was greater than that for the ownership statement, and 64% of participants had an RHI index value greater than their response to the ownership statement. In comparison, only 37% of participants had an RHI index value greater than their response to the ownership statement in the asynchronous condition. The percentage of participants reporting positive responses was generally greater for this dataset, probably due to the different rating system used for questionnaire responses.

In the data presented by Motyka and Litwin (2019), responses to referral of touch were not statistically significantly greater than those for ownership in the synchronous condition (medians = 1.5 vs. 1, *W* = 343.5, *p* = 0.871, *r* = 0.0315, 95% CI = [−0.330, 0.385]), with only 36% of participants displaying an effect in this direction (Figure 1). There was no statistically significant difference between referral of touch and ownership statements in the asynchronous condition (both medians = −2, *W* = 133, *p* = 0.0634, *r* = −0.389, 95% CI = [−0.679, 0.00670]), or when comparing the difference between the synchronous and asynchronous conditions (medians = 3 vs. 1.5, *W* = 584, *p* = 0.0933, *r* = −0.0839, 95% CI = [−0.382, 0.230]).

Responses to the referral of touch and ownership statements were positively correlated in the synchronous condition (*r*_τ_ = 0.513, 95% CI = [0.359, 0.667], *p* < 0.001, Figure 2), and when examining the difference between synchronous and asynchronous conditions (*r*_τ_ = 0.490, 95% CI = [0.338, 0.641], *p* < 0.001). The percentage of participants with a positive response to referral of touch was matched with that for ownership (66%; Table 1). In addition, the percentage of participants with a positive response for the RHI index (68%) was greater than that for the ownership statement, though only 36% of participants had an RHI index value greater than their response to the ownership statement. In comparison, only 16% of participants had an RHI index value greater than their response to the ownership statement in the asynchronous condition.

Combining our dataset with that of Motyka and Litwin (2019), who had a comparable scoring system, provided an assessment of responder ratio in 109 individuals (Table 2). As one would expect from the estimates reported for individual articles (Table 1), 61% of individuals (*n* = 67) reported an experience of ownership over the false hand, whilst a greater number (70%) reported referral of touch (*n* = 76). 87% of people who reported ownership also reported referral of touch (*n* = 58/67). Notably, those who reported referral of touch but not ownership made up around 43% of ownership non-responders (*n* = 18/42), indicating that reports for these two subjective experiences do not always overlap. Interestingly, there was also evidence for an extreme minority (8%, *n* = 9) that reported a feeling of ownership over the false hand without referral of touch.

**Table 2:** Contingency table for combined synchronous datasets (Motyka and Litwin, 2019; Reader et al., 2020).

		**Referral of touch**		**Total**
		**No**	**Yes**	
**Ownership**	**No**	24	18	42
	**Yes**	9	58	67
**Total**		33	76	109

In contrast to responses in the synchronous condition, in the asynchronous condition the majority (75%) of participants reported neither referral of touch nor a feeling of ownership (*n* = 82), in keeping with this condition being an experimental control (Table 3). Positive response rates to the two phenomena were low, with 15% of individuals (*n* = 16) reporting referral of touch and 20% reporting a feeling of ownership over the false hand (*n* = 22). Interestingly, the number of individuals positively reporting a sense of ownership without referral of touch was comparable to the number of individuals reporting both phenomena (10%, *n* = 11 in both cases).

**Table 3:** Contingency table for combined asynchronous datasets (Motyka and Litwin, 2019; Reader et al., 2020).

		**Referral of touch**		**Total**
		**No**	**Yes**	
**Ownership**	**No**	82	5	87
	**Yes**	11	11	22
**Total**		93	16	109

When examining the distribution of positive responses across synchronous and asynchronous conditions combined (Supplementary Figure 1), we observed that 37% of participants only affirmed referral of touch and ownership in the synchronous condition (*n* = 40). 19% of participants did not affirm referral of touch or ownership in either condition (*n* = 21) and 15% of participants affirmed only referral of touch in the synchronous condition (*n* = 16). The rest of the participants were distributed across the rest of the response combinations.

**Figure 3:**
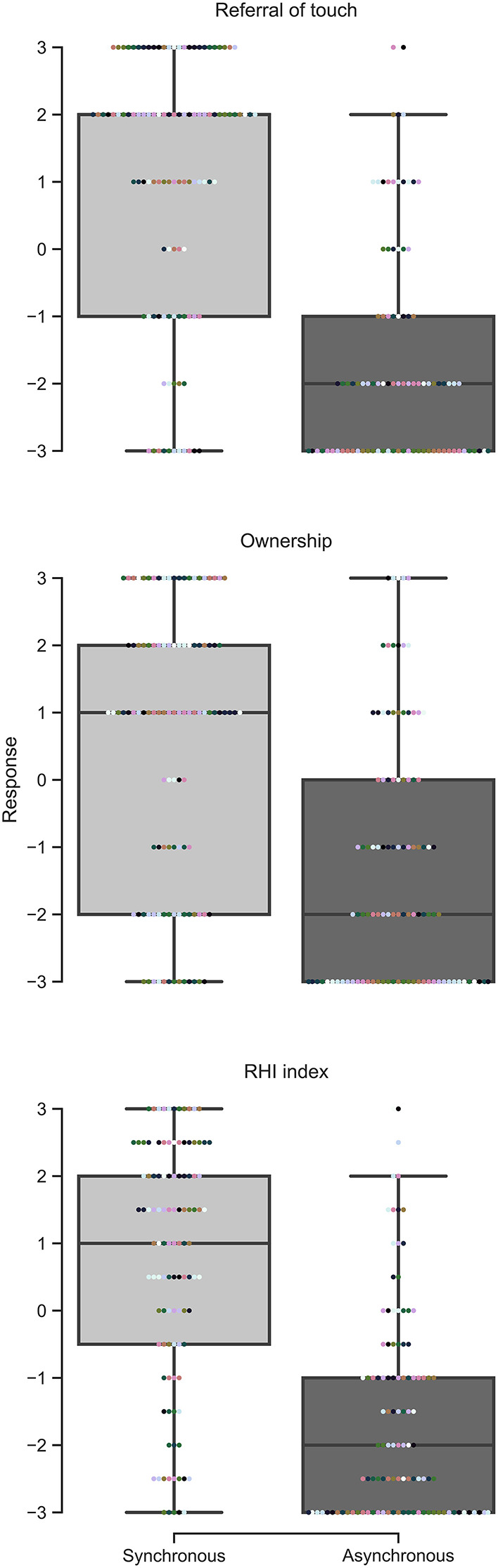
Questionnaire responses for the combined analysis.

As one would expect, responses to the referral of touch statement were more positive in the synchronous condition compared to the asynchronous condition (*W* = 4,570.5, *p* < 0.001, *r* = 0.884, 95% CI = [0.823, 0.925]) (Figure 3). Similar results were observed for the ownership statement (*W* = 3,277.5, *p* < 0.001, *r* = 0.836, 95% CI = [0.745, 0.897]) and for the RHI index (*W* = 4,969, *p* < 0.001, *r* = 0.892, 95% CI = [0.836, 0.929]) (Figure 3).

## Discussion

We examined referral of touch and the feeling of ownership in the RHI in three freely available datasets. We observed five key findings. First, referral of touch tends to be reported more strongly than the feeling of ownership over the false hand. Second, affirmation of referral of touch is more common than affirmation of ownership. Third, a greater number of individuals are found to have a positive response to an RHI index compared to ownership alone, and this increased value may be observed in at least a third of participants. Fourth, those who report referral of touch but not ownership make up almost half of ownership non-responders. Lastly, most participants who report ownership also report referral of touch, and the two perceptual phenomena are moderately positively correlated. This correlation was replicated in all three studies and is consistent with multisensory models of body ownership.

That referral of touch is reported more strongly than the feeling of ownership, and that more participants report referral of touch, backs up evidence from the earliest RHI experiments (Botvinick and Cohen, 1998; Holmes and Spence, 2007). Whilst ~60–80% of participants report referral of touch, less (~50–70%) report a feeling of ownership over the false hand, in keeping with previous studies (e.g., Kalckert and Ehrsson, 2014; Kalckert et al., 2019). One possibility is that these results reflect differences in decision criteria or response bias rather than genuine differences in perceptual awareness, such that participants require different levels of evidence to accept the statements. Participants may be more conservative in expressing their support for the ownership statement, since this is strongly at odds with existing beliefs and experiences of the real body. However, our combined analysis revealed that in the synchronous condition, 87% of people who positively responded to the ownership statement also reported referral of touch. These results broadly support the idea that referral of touch may precede the development of ownership over the false hand (Makin et al., 2008), the latter of which does not occur in every individual and likely requires experiencing proprioceptive sensations from the false hand. This is in keeping with a multisensory model of body ownership that views referral of touch and ownership as two aspects of the formation of a coherent multisensory representation of the (false) hand as one's own (Kilteni et al., 2015; Samad et al., 2015; Ehrsson, 2020; Litwin, 2020). Namely, tactile and proprioceptive information from the real hand are combined with visual information of the false hand to create the experience of ownership. Accordingly, and in keeping with previous research (Longo et al., 2008), reports of the two sensations are moderately correlated, and the strength of this correlation was consistent across three separate datasets when comparing statements in the synchronous condition (*r*_τ_ = 0.513–0.607) or the difference between synchronous and asynchronous conditions (*r*_τ_ = 0.381–0.580). Generally, the results of our re-analysis indicate that referral of touch, reflecting the unification of visual and tactile perception in hand-centered space, may indeed be very important for developing a sense of ownership over the false hand during the illusion.

However, there was also evidence for a minority of participants who reported experiencing ownership over the false hand without affirming referral of touch in our combined analysis (9/109 in the synchronous condition, and 11/109 in the asynchronous condition). It is possible that these individuals place greater weight on visuoproprioceptive than visuotactile feedback, since visuoproprioceptive integration has also been linked to ownership sensations (Walsh et al., 2011; Kalckert and Ehrsson, 2012; Samad et al., 2015). This would suggest that referral of touch is not a necessary condition for developing a sense of ownership in the RHI, but rather any combination of at least two sensory modalities (e.g., vision and proprioception). Another possibility is that these responses reflect some level of participant suggestibility (Marotta et al., 2016), which have been found to predict some variance (<10%) in participant questionnaire responses across conditions and statement types in the RHI (Lush et al., 2020). Of course, this claim is based on the assumption that referral of touch is a necessary condition for ownership in the RHI, which may be unfounded. Nevertheless, the role of suggestibility in the RHI is worth mentioning, since it may alter the interpretation of our findings if some statements are influenced by suggestibility and others are not. However, Marotta et al. (2016) observed that even participants low in suggestibility showed greater responses to referral of touch and ownership in the synchronous condition compared to the asynchronous condition, with stronger affirmation for referral of touch than ownership.

Our findings also have important implications for the performance and interpretation of RHI experiments. In keeping with Kalckert et al. (2019), combining referral of touch and ownership statements results in a greater percentage of individuals ‘reporting’ a positive response, with potentially over half of participants ending up with a higher RHI index value compared to their ownership response. We build on previous findings by showing that those who report referral of touch but not ownership may make up almost half of ownership non-responders. Overall, these results suggest that averaging these different statements may be suboptimal if one is specifically interested in using the RHI to examine body ownership. Whilst we found that effect sizes between the synchronous and asynchronous conditions were comparable for the ownership statement and RHI index, this analysis was performed on a considerably bigger sample size than most studies using the RHI (*n* = 109). To contrast, in our own data (*n* = 59) the same analysis results in an effect size of *r* = 0.119 for the ownership statement, *r* = 0.532 for referral of touch, and *r* = 0.685 for the RHI index, suggesting that in some cases it may not be appropriate to assume the RHI index captures body ownership sensations effectively. Using an RHI index could therefore possibly result in a poor estimation of any effects that are purported to be associated specifically with the subjective affirmation of ownership of the false hand. For instance, correlations observed between the magnitude of an RHI index and another variable may represent a relationship with referral of touch rather than the body ownership experience.

In addition to reporting correlations with an RHI index, we suggest that it can often be valuable to examine correlations for the ownership and referral of touch statements in isolation, to explore which statement is driving the overall result. This can be particularly important in studies where there could be reasons to expect that ownership and referral of touch might not go “hand in hand,” like experiments with non-corporeal objects (e.g., balloons and blocks of wood) or tool use, or in experiments where people might ‘mistakenly’ affirm ownership based on mirror recognition or agency mechanisms (but without a genuine perceptual body illusion). We also suggest that authors studying the RHI should publish the full results of questionnaires, including all individual statements (at least as supplementary material and ideally also freely available), and not only report average scores based on groups of statements, so that referral of touch and feeling of ownership (and potentially other experiences) can be independently assessed.

Finally, we must consider the limitations of our experimental approach. In our original experiment (Reader et al., 2020), we used a single statement to assess referral of touch (“It seemed like the touch I felt was caused by the brush touching the rubber hand”). However, a second statement is often used in addition to this one (e.g., “It seemed like I was feeling the touch of the brush in the location where I saw the rubber hand being touched”). We chose not to include this statement when generating an RHI index in additional datasets to maintain consistency with the analysis of our own results. This may mean it is harder to gauge the degree to which this statement may play a role in the generation of an RHI index. However, it appears that combining both statements along with the ownership statement to create an RHI index may result in an even larger percentage of ‘positive’ responses (Supplementary Material, Table 1). The ‘location of touch’ statement shows greater affirmation than the ‘causal’ referral of touch statement, as evident when these statements are directly compared for the synchronous condition in Supplementary Material (though not when considering the difference between synchronous and asynchronous conditions). Although in the synchronous condition, and when considering the difference scores between the synchronous and asynchronous conditions, the two referral of touch statements were correlated (Supplementary Material), some have previously reported that the two statements are not statistically related (Longo et al., 2008). Furthermore, and again in contrast to the findings of Longo et al., the location of touch statement was correlated with the ownership statement, both when analyzing the synchronous condition scores and when considering the difference score between the synchronous and asynchronous conditions. Thus, the current results suggest that the ‘location of touch’ and ‘causal’ sensations may both be related to the feeling of ownership over the false hand in the RHI. Nevertheless, separating these statements when reporting questionnaire results may be prudent, as emphasized in general above.

Interpreting our findings may also be limited by differences between experimental paradigms used in the three studies we analyzed. For example, there was variation in the distance between the real and false hands, the scoring system for questionnaires, and the duration of illusion induction. These factors could explain why the results of Motyka and Litwin (2019) tended to diverge from those of Engelen et al. (2017) and our own experiment. For instance, responses to referral of touch and ownership statements were not significantly different in the data from Motyka and Litwin (2019), whilst the other datasets showed stronger responses to referral of touch. This could be because we combined data from Motyka and Litwin (2019) in which the real and false hands were separated by different distances (8 and 24 cm), which may have led to different relationships between referral of touch and ownership. However, neither group displayed a significant difference between referral of touch and ownership statements, and both groups displayed a significant difference between synchronous and asynchronous conditions for the two statements, suggesting that they both tended to experience the illusion. It is possible that with a larger sample size, differences between referral of touch and ownership ratings might have been observed by Motyka and Litwin (2019). However, correlation results for Motyka and Litwin (2019) were similar to those of the other datasets we analyzed, which suggests that these may be the more robust of our findings. Whilst the impact of methodological differences on RHI experiments certainly requires further study (Riemer et al., 2019), the differences between the three experiments were not vast (e.g., none were using virtual reality or a different form of multisensory stimulation), and we believe that participants were likely to be reporting similar experiences of referral and touch and ownership in the three experiments discussed here.

In conclusion, we provide a detailed examination of referral of touch and the feeling of ownership in the RHI. These results build on previous observations that referral of touch is more strongly and consistently reported than the feeling of ownership in the RHI paradigm, and demonstrate that referral of touch and ownership are correlated during this classic illusion. In addition, our results suggest that averaging these two types of statement should be considered with caution, especially in paradigms where referral of touch and ownership might be expected to deviate and/or when one is specifically interested in characterizing explicit reports of ownership, and done only when justified by the experimental hypothesis and research question.

## Data Availability Statement

Publicly available datasets were analyzed in this study. This data can be found here: https://doi.org/10.17605/OSF.IO/NYHZQ, https://doi.org/10.1371/journal.pone.0186009, and https://doi.org/10.1177/0301006619865189.

## Ethics Statement

The authors study involving human participants was reviewed and approved by the Swedish Ethical Review Authority. The participants provided their written informed consent to participate in this study.

## Author Contributions

AR and VT collected and analyzed the data. All authors contributed to writing the manuscript.

## Conflict of Interest

The authors declare that the research was conducted in the absence of any commercial or financial relationships that could be construed as a potential conflict of interest.
