# Supplementary material for: The Relationship Between Referral of Touch and the Feeling of Ownership in the Rubber Hand Illusion
Source: Front Psychol. 2021 Feb 11;12:629590. doi: 10.3389/fpsyg.2021.629590 (PMC7904681; doi:10.3389/fpsyg.2021.629590)
Supplement: Supplementary file 1 [file Data_Sheet_1.PDF]

Supplementary Material: The relationship between referral of touch and the feeling of ownership in the rubber hand illusion

Arran T. Reader, Victoria S. Trifonova, and H. Henrik Ehrsson

Comparison of two referral of touch statements:

In the synchronous condition responses to the 'location of touch' statement were greater than those to the 'causal' statement for both Engelen et al. (2017) (medians = 6 versus 5.57, $W=844.5$, $p<.001$, $r=-.0458$) and Motyka and Litwin (2019) (medians = 2 versus 1.5, $W=387.5$, $p=.0177$, $r=-.392$). Similar results were observed for the asynchronous condition for Motyka and Litwin (2019) (both medians = -2, $W=164.5$, $p=.0245$, $r=-.742$) but not Engelen et al. (2017) (both medians = 2, $W=706$, $p=.134$, $r=-.202$). We also compared the difference between synchronous and asynchronous conditions across the two referral of touch statements. In the data from Engelen et al. (2017) we observed that there was no significant difference between the two statements ($W=771.5$, $p=.806$, $r=-.128$). A similar result was observed in the data from Motyka and Litwin (2019) ($W=359$, $p=.684$, $r=-.437$).

In the synchronous condition the two referral of touch statements were correlated in the datasets of both Engelen et al. (2017) ($r_\tau=.548$, $p<.001$) and Motyka and Litwin (2019) ($r_\tau=.451$, $p<.001$). In addition, the difference between the synchronous and the asynchronous conditions was correlated across the two referral of touch statements in the data from Engelen et al. (2017) ($r_\tau=.401$, $p<.001$) and Motyka and Litwin (2019) ($r_\tau=.521$, $p<.001$) (Supplementary Figure 2).

The 'location of touch' statement was correlated with the ownership statement in the synchronous condition for Engelen et al. (2017) ($r_\tau=.426$, $p<.001$) and Motyka and Litwin (2019) ($r_\tau=.405$, $p<.001$). This was true also when correlating the difference between the synchronous and asynchronous conditions for Engelen et al. (2017) ($r_\tau=.436$, $p<.001$) and Motyka and Litwin (2019) ($r_\tau=.288$, $p=.00823$) (Supplementary Figure 3).

Supplementary Table 1: Analysis using both referral of touch statements to create an RHI index

Article	Ownership statement	Referral of touch statements	Positive response to mean of referral of touch statements (%)	Positive response to ownership statement (%)	Positive response for RHI index (%)	Participants with RHI index value greater than ownership value (%)
Engelen et al. (2017)	"I felt as if the rubber hand were my hand"	"It seemed as if I were feeling the touch of the finger in the location where I saw the rubber hand touched", "It seemed as though the touch I felt was caused by the finger touching the rubber hand"	92	76	88	78
Motyka & Litwin (2019)	"I felt as if the rubber hand were my hand."	"It seemed as if I were feeling the touch of the paintbrush in the location where I saw the rubber hand touched", "It seemed as though the touch I felt was caused by the paintbrush touching the rubber hand"	76	66	76	50

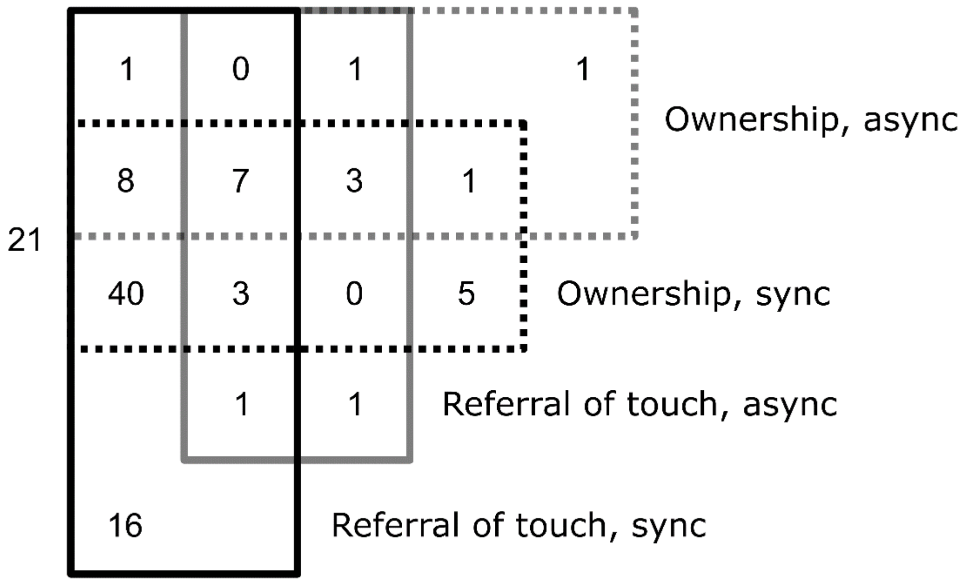

Supplementary Figure 1: Affirmation of referral of touch and ownership across all condition combinations (numbers of participants)

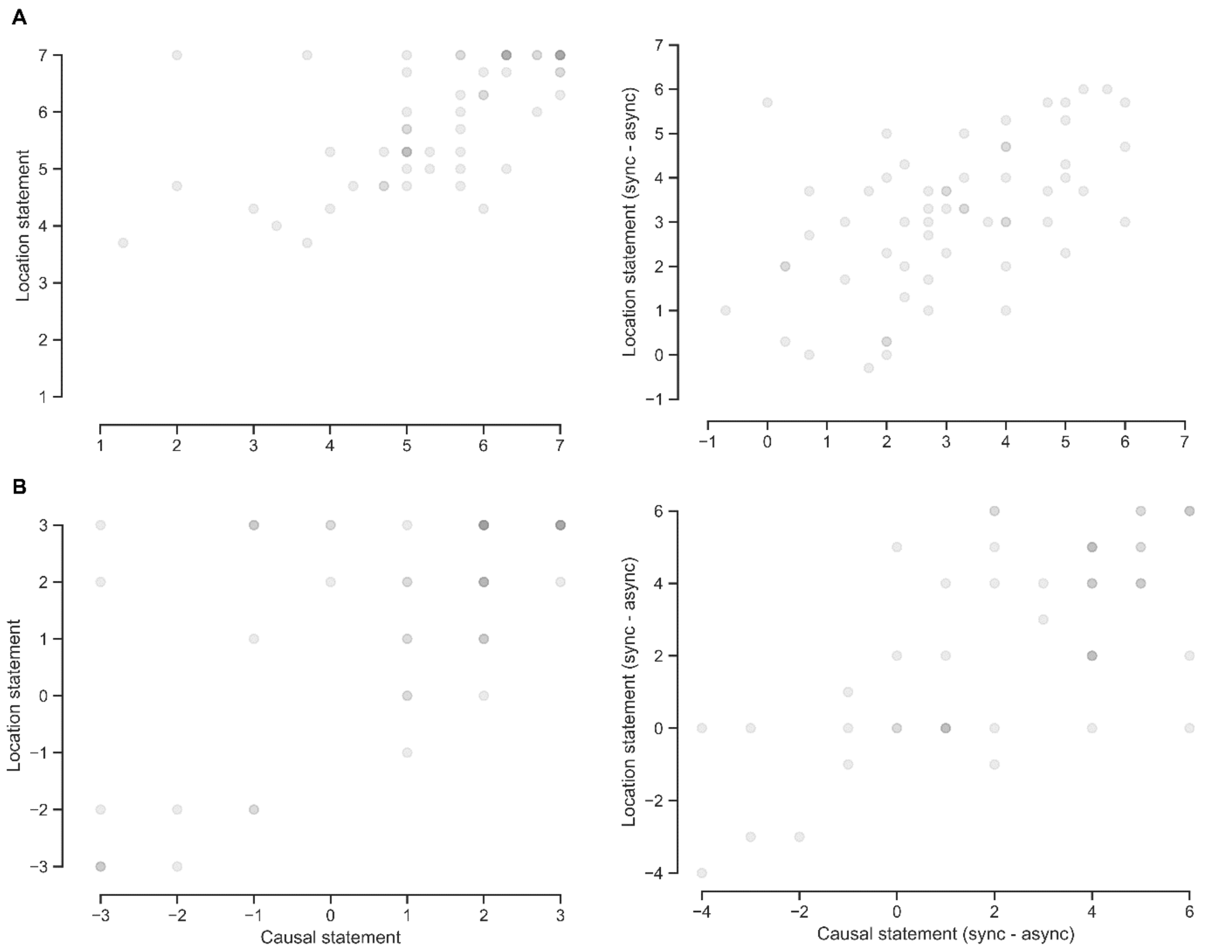

Supplementary Figure 2: Scatterplots comparing responses to the two referral of touch statements

Data are displayed for A) Engelen et al. (2017), and B) Motyka and Litwin (2019).

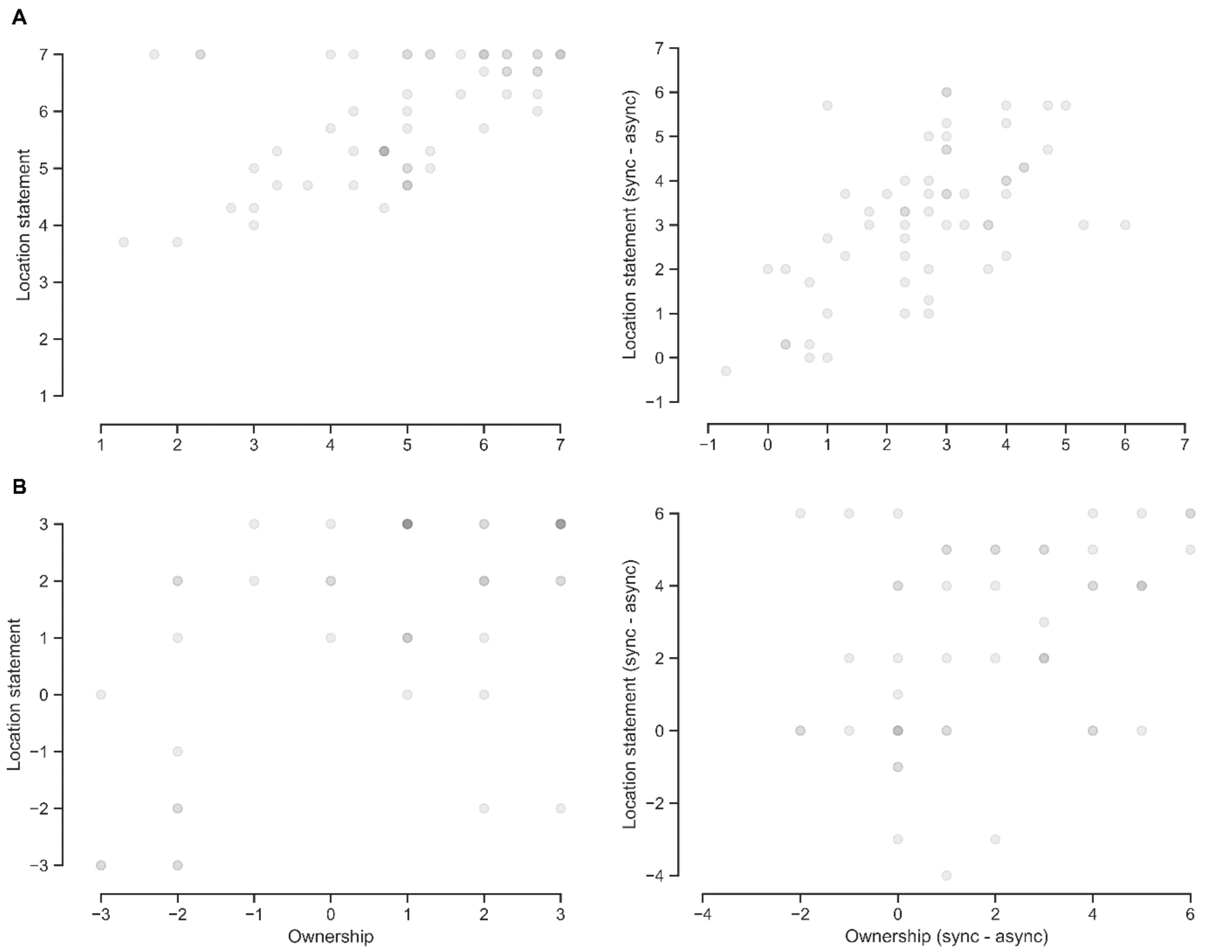

Supplementary Figure 3: Scatterplots comparing responses to the 'location of touch' statement and the ownership statement

Data are displayed for A) Engelen et al. (2017), and B) Motyka and Litwin (2019).
